# Different exercise modalities have distinct effects on the integrin-linked kinase (ILK) and Ca^2+^ signaling pathways in the male rat bone

**DOI:** 10.14814/phy2.12568

**Published:** 2015-10-14

**Authors:** Dharani M Sontam, Elwyn C Firth, Peter Tsai, Mark H Vickers, Justin M O’Sullivan

**Affiliations:** 1 The Liggins Institute, University of AucklandAuckland, New Zealand; 2 Gravida: National Centre for Growth and Development, University of AucklandAuckland, New Zealand; 3 Department of Sport and Exercise Science, University of AucklandAuckland, New Zealand; 4 School of Biological Sciences, University of AucklandAuckland, New Zealand

**Keywords:** Cortical bone, exercise, gene expression, mechanotransduction, RNA-Seq

## Abstract

Mechanical loading is essential to maintain optimal skeletal health. Despite the fact that early-life exercise has positive, long-lasting effects on the musculo-skeletal system, the response of the musculo-skeletal system to spontaneous low-impact exercise has been poorly studied. Previously, we identified subtle morphological changes in the femoral diaphysis of exercised animals compared to nonexercised controls. We hypothesized that significant changes in gene expression of cells should precede significant measurable phenotypic changes in the tissues of which they are part. Here, we employed RNA-Seq to analyse the transcriptome of the cortical bone from the femoral mid-diaphysis of prepubertal male Sprague-Dawley rats that were assigned to control (CON); bipedal stance (BPS); or wheel exercise (WEX) groups for 15 days. We identified 808 and 324 differentially expressed transcripts in the BPS and WEX animals respectively. While a number of transcripts change their levels in an exercise-specific manner, we identified 191 transcripts that were differentially expressed in both BPS and WEX. Importantly, we observed that the exercise mode had diametrically opposite effects on transcripts for multiple genes within the integrin-linked kinase (ILK) and Ca^2+^ signaling pathways such that they were up-regulated in BPS and down-regulated in WEX. The findings are important for our understanding of possible ways in which different exercise regimens might affect bone when normal activities apply mechanical stimuli during postnatal growth and development.

## Introduction

Associational studies have established positive and long-lasting effects of early-life exercise in people who engaged in competitive sport as youths or adolescents (Kannus et al. 1995; Karlsson et al. 1995). Due to the intense level(s) of training required to achieve this competitive rank, such activity is unsuitable as a health recommendation for a majority of any community. However, despite the fact that mild or moderate early-life exercise does not negatively affect bone (Firth et al. 2004) or joint development (Van Weeren and Barneveld 1999; Jones et al. 2003), the long-lasting effects of this form of exercise on the musculoskeletal and other body systems have been poorly described. Indeed, morphological studies into the effects of early exercise are further complicated by the fact that the effects on bone phenotype in young animals might be obscured by the normal musculoskeletal growth, and thus be difficult to detect.

Integrating physiological, morphological, and molecular responses of bone to spontaneous exercise is vital for exercise to be advocated as an effective strategy for maintaining/improving bone health parameters. Previously, we identified subtle morphological changes in the femoral diaphysis of prepubertal male rats that were allowed to undertake wheel running (WEX) or had to rise to an erect bipedal stance (BPS) when feeding, for a period of only 15 days (Sontam et al. 2015). These exercise programs were chosen to minimize the confounding effects of stress, and the exercise period was limited to 15 days to ensure that the manipulation was specific to the prepubertal period.

We hypothesized that significant changes in gene expression of cells should precede significant measurable phenotypic changes in the tissues of which they are part. Here, we characterize and compare the global gene expression profile changes in the diaphysis of BPS or WEX rats to those of conventionally housed rats (Control; CON). We identify shared and exercise-specific changes to pathways involved in mechanotransduction, energy homeostasis and cell-cell junction signaling. We show diametrically opposite effects on transcripts for multiple genes within the integrin-linked kinase (ILK) and Ca^2+^ signaling pathways such that they were up-regulated in BPS and down-regulated in WEX.

## Materials and Methods

### Study design

Tissues that were analysed in this paper were collected simultaneously with those reported in Sontam et al. (Sontam et al. 2015). All animal work was performed under approval R1068 (Animal Ethics Committee, University of Auckland). Twenty-four weanling (21 day old) male pups were obtained from four time-mated Sprague-Dawley rats and assigned to three exercise groups (*n* = 8): control (CON), bipedal stance (BPS), and wheel exercise (WEX) as previously described (Sontam et al. 2015). Briefly, body composition of the animals was determined before the start of exercise, on day 20 (D_20_) using dual energy X-ray absorptiometry (DXA, Lunar Hologic, GE, Waltham, MA). The animals were assigned to one of the three exercise groups such that the body weight and total fat percentage were not significantly different between the groups at the outset of the experimental protocol. All animals had ad libitum access to food (Diet 2018, Teklad Global 18% Protein Rodent Diet, Harlan, Teklad) and water.

All groups were pair-housed. CON group rats had unrestricted movement within their cage. BPS animals had to fully extend their tibiotarsal and femorotibial joints to reach their food which was 220 mm above the cage floor. WEX rats could voluntarily access a zero-resistance activity wheel. Food and water intake was measured daily from D_20–27_ and on alternative days from D_27–35_. The trial period lasted from D_21_ to D_35_. A further DXA scan was undertaken at the end of the exercise period (D_35_).

### Tissue collection

On D_36_, animals were culled by decapitation under isoflurane anesthesia following an overnight fast. Epididymal and retroperitoneal fat pads were collected and weighed. The left femur was immediately dissected from the surrounding soft tissue and was sectioned into proximal, middle, and distal thirds, which were snap-frozen in liquid nitrogen and stored at −80°C; the right femur was harvested, cleaned, and stored in 70% ethanol for micro-computed tomography (*μ*CT) analysis.

### RNA extraction

From the mid-femoral section, total RNA was extracted according to Ayturk et al. (Ayturk et al. 2013). Briefly, bone marrow was removed from the mid-diaphysis by centrifugation (10,000 rpm, 2 min, 4°C). The bone was then crushed into a fine powder and transferred to a 1.5 mL sterile microcentrifuge tube containing 1 mL of TRIzol® Reagent (#15596-026, Life Technologies, Carlsbad, CA), and homogenized by sonication (Bandelin Sonopuls HD2070, Bandelin, Berlin, Germany). Total RNA was extracted using TRIzol®-chloroform extraction and cleaned using the RNeasy Mini Kit (#74104, Qiagen, Hilden, Germany). Traces of genomic DNA were removed by on-column DNA digestion (RNase-free DNase Set; Qiagen). RNA quality/quantity was measured using spectrophotometry (Nanodrop ND-1000, Thermo Scientific, Wilmington, DE) and Bioanalyzer (Model 200, Agilent Technologies, Santa Clara, CA). All sample RNA integrity numbers (RIN) numbers ranged from 6.1 to 8.7 (Table1). Purified RNA was stored at −80°C until sequencing (50 bp, paired end; BGI China). The sequence data have been deposited in NCBI’s Gene Expression Omnibus (GEO; (Edgar et al. 2002)) and are accessible through GEO Series accession number GSE67787 (http://www.ncbi.nlm.nih.gov/geo/query/acc.cgi?acc= GSE67787).

**Table 1:** Alignment summary of RNA-seq reads to the rat transcriptome

Treatment	Replicate	RIN	Total read (pairs)	Aligned pairs	Concordant pair alignment rate (%)	Multiple alignments (%)	Discordant alignments (%)
CON	AA11	7.9	16180254	15354030	93.2	8.6	1.7
	AA22	8.2	21510189	20408251	93.3	8.6	1.7
	AA33	8.7	19985990	19004887	93.7	8.6	1.5
	AA44	7.1	18219920	17249629	92.9	7.6	1.8
BPS	BB11	7.7	26109589	24724072	93.2	8.3	1.6
	BB22	8.3	22931245	21753771	93.3	8.3	1.6
	BB33	7.4	30278018	28563380	93.0	7.3	1.4
	BB44	6.1	30108924	28280303	92.6	7.3	1.5
	BB55	6.1	37307805	35225824	93.2	7.0	1.2
WEX	CC11	8.3	12893153	12254899	93.5	8.8	1.7
	CC22	7.5	18013539	17176184	93.9	8.9	1.5
	CC33	7.5	18802378	17921117	93.9	7.9	1.4
	CC44	8.2	20776702	19714361	93.4	8.4	1.6
	CC55	7.7	19575453	18607230	93.5	7.9	1.6

### Read alignment and differential gene expression analysis

Four biological replicates from CON and five biological replicates each from BPS and WEX groups were included in the differential gene expression analysis. Sequenced read quality was assessed (FastQC; (http://www.bioinformatics.babraham.ac.uk/projects/fastqc/)). Phred quality scores were >28 and trimming was not necessary. Sequenced reads were aligned to the rat transcriptome (reference sequence NCBI version Rnor_5.0) using TopHat (version 2.0.12) by providing the GTF file and parameters – r 200 -G (Trapnell et al. 2009). The concordant pair alignment rate was 92.6–93.9% with 7.0–8.9% multiple alignments and 1.2–1.7% discordant alignment rates.

Differential gene expression was determined (Cufflinks version 2.2.1) (Trapnell et al. 2010). Cuffdiff was run with the parameters – u (multiread correction algorithm) and – b (bias detection and correction algorithm). Differentially expressed transcripts were subjected to term enrichment analysis using GOTermFinder (http://go.princeton.edu/cgi-bin/GOTermFinder) (Boyle et al. 2004). The *P*- value cut-off was set at 0.05 and gene lists queried against the Rat Genome Database (RGD).

### Pathway analysis

Differentially expressed transcripts were loaded into Ingenuity Pathway Analysis (IPA Winter Release 2014, IPA, QIAGEN Redwood City, www.qiagen.com/ingenuity). The dataset was filtered using the following attributes: *P*-value = 0.05; the user dataset was used as the reference set; and *Rattus norvegicus* was specified as the species. IPA identified pathways with significant enrichments and ranked them according to *P*-values derived from a right tail Fisher exact test.

## Results

The phenotypic characteristics of animals within the BPS, CON, and WEX groups have been previously described by our group (Sontam et al. 2015). Briefly, we showed that the diaphysis of femurs within the WEX group had a significantly higher cortical cross-sectional thickness and percent closed porosity than the CON group (Sontam et al. 2015). We showed that the effects of exercise on these changes were independent of caloric intake, body weight, or measures related to stress. The initial changes that we saw led us to hypothesize that RNA expression patterns may be altering in response to the different exercise modalities. Therefore, we undertook a retrospective analysis of the RNA profiles in the femurs of these animals.

### Differential gene expression

There were 324 differentially expressed transcripts in the mid-diaphysis of the WEX animals when compared to CON (False Discovery Rate [FDR] adjusted *P*-value < 0.05). The gene transcripts that were up-regulated in the WEX rats were enriched for Gene Ontology (GO) terms associated with skeletal system development and homeostasis (*P*-value ≤ 0.0218; Table2A). These GO terms included genes that are involved in bone metabolism (*e.g*. *Bmp2, Cat, Dkk3, Ephb3, Epha4, Fap, Fzd1, Gdf10, Gnas, Lrrc17, Myoc, Nfib, Ostn, Tbx18, Twist1,* and *Wnt5a*). The gene transcripts that were down-regulated in the mid-diaphysis of the WEX rats were enriched for GO terms associated with muscle and blood (*P*-value ≤ 6.44E-07; Table2B).

**Table 2:** GOtermfinder results for (A) up-regulated genes and (B) down-regulated genes in response to WEX

ID	Term	*P*-value	Annotated genes
(A)			
GO:0050673	Epithelial cell proliferation	4.51E-04	*Fap, Col8a2, Nfib, Tbx18, Wnt5a, Eya1, Bmp2, Twist1, Tnmd, Col8a1*
GO:0001649	Osteoblast differentiation	8.37E-04	*Cat, Myoc, Fzd1, Ostn, Gnas, Bmp2, Twist1, Gdf10*
GO:0003151	Outflow tract morphogenesis	2.80E-03	*Wnt5a, Eya1, Sema3c, Twist1, Fzd1*
GO:0045667	Regulation of osteoblast differentiation	3.47E-03	*Gnas, Bmp2, Twist1, Fzd1, Gdf10, Ostn*
GO:0030278	Regulation of ossification	5.15E-03	*Wnt5a, Gnas, Bmp2, Twist1, Fzd1, Gdf10, Ostn*
GO:0001501	Skeletal system development	7.05E-03	*Myoc, Nfib, Ostn, Gnas, Wnt5a, Eya1, Bmp2, Twist1, Lrrc17, Hapln1*
GO:0001503	Ossification	8.18E-03	*Cat, Myoc, Fzd1, Ostn, Gnas, Wnt5a, Bmp2, Twist1, Gdf10*
GO:0090090	Negative regulation of canonical Wnt signalling pathway	2.18E-02	*Wnt5a, Bmp2, Fzd1, Dkk3, Tbx18*
GO:0035295	Tube development	2.25E-02	*Cat, Sema3c, Fzd1, Nfib, Epha4, Tbx18, Wnt5a, Eya1, Ephb3, Bmp2, Twist1*
GO:0010811	Positive regulation of cell-substrate adhesion	2.41E-02	*Ndnf, Myoc, Edil3, Col8a1, Ccl21*
(B)			
GO:0006936	Muscle contraction	4.74E-13	*Myl2, Actc1, Tmod4, Actn3, Myom2, Ttn, Stac3, Cacna1s, Tnni1, Mybpc1, Tnnc1, Pgam2, Actn2, Tnnt1, Myl3, Ncf1, Hspb6, Myh7, Cav3, Scn4b, Mybpc2, Rcsd1, Myom1, Mylk2, Ryr1*
GO:0003012	Muscle system process	1.33E-12	*Myl2, Actc1, Tmod4, Actn3, Myom2, Ttn, Stac3, Cacna1s, Hrc, Tnni1, Mybpc1, Tnnc1, Pgam2, Actn2, Tnnt1, Myl3, Ncf1, Hspb6, Myh7, Trim72, Cav3, Scn4b, Mybpc2, Rcsd1, Myom1, Mylk2, Ryr1*
GO:0030218	Erythrocyte differentiation	5.80E-09	*Trim10, Gfi1b, Rhd, Bcl6, Dmtn, Ank1, Inpp5d, LOC287167, Tal1, Rhag, Alas2, Hmgb2, Isg15, Klf1, Ahsp*
GO:0034101	Erythrocyte homeostasis	2.31E-08	*Trim10, Gfi1b, Rhd, Bcl6, Dmtn, Ank1, Inpp5d, LOC287167, Tal1, Rhag, Alas2, Hmgb2, Isg15, Klf1, Ahsp*
GO:0061061	Muscle structure development	5.07E-08	*Jph2, Myl2, Actc1, Tmod4, Kel, Ankrd23, LOC100910104, Flnc, Ttn, Pax5, Stac3, Cacna1s, Trdn, Srpk3, Hist1h1b, Smtnl1, Tnni1, Tnnc1, Myl3, Ankrd2, Rbm38, Capn3, Myh7, Trim72, Cav3, Mypn, Mylk2, Ryr1, Ldb3*
GO:0055002	Striated muscle cell development	1.85E-07	*Myl2, Actc1, Tmod4, Capn3, Kel, Cav3, Ankrd23, LOC100910104, Flnc, Ttn, Mypn, Stac3, Cacna1s, Ryr1, Ldb3*
GO:0051146	Striated muscle cell differentiation	3.14E-07	*Myl2, Actc1, Tmod4, Kel, Ankrd23, LOC100910104, Flnc, Ttn, Stac3, Cacna1s, Trdn, Rbm38, Ankrd2, Capn3, Trim72, Cav3, Mypn, Ryr1, Ldb3*
GO:0002262	Myeloid cell homeostasis	3.35E-07	*Trim10, Gfi1b, Rhd, Bcl6, Dmtn, Ank1, Inpp5d, LOC287167, Tal1, Rhag, Alas2, Hmgb2, Isg15, Klf1, Ahsp*
GO:0030239	Myofibril assembly	4.95E-07	*Myl2, Actc1, Tmod4, Capn3, Cav3, Ankrd23, LOC100910104, Ttn, Mypn, Ldb3*
GO:0055001	Muscle cell development	6.44E-07	*Myl2, Actc1, Tmod4, Capn3, Kel, Cav3, Ankrd23, LOC100910104, Flnc, Ttn, Mypn, Stac3, Cacna1s, Ryr1, Ldb3*

There were 808 differentially expressed transcripts (DETs) in the mid-diaphysis of the BPS animals when compared to CON (False Discovery Rate [FDR] adjusted *P*-value < 0.05). The gene transcripts that were up-regulated in the BPS group were associated with GO terms related to muscle cells and energy metabolism (*P*-value ≤ 5.10E-18; Table3A). The gene transcripts that were down-regulated in the BPS animals were enriched for GO terms associated with the immune system (*P*-value ≤ 5.20E-12; Table3B).

**Table 3:** GOtermfinder results for (A) up-regulated genes and (B) down-regulated genes in response to BPS

ID	Term	*P*-value	Annotated genes
(A)			
GO:0061061	Muscle structure development	2.05E-30	*Ank2, Jph2, Myod1, Mamstr, Slc25a4, Flnc, Chrnb1, Cacna1s, Acadm, Neurl1, Myl3, Bin1, Six1, Klhl41, Wnt5a, Sgcb, Ryr1, Lmod1, Nr4a1, Myh14, Meox2, Myh11, P2rx5, Lgals1, Srpk3, Trdn, Klhl40, Jph1, Ppapdc3, Mylpf, Sgca, Xirp2, Nmrk2, Eln, RGD1309821, Myog, Cfl2, Itga8, Nexn, Casq2, Myl2, Col14a1, Actc1, Fzd1, Sgcg, Csrp2, Smyd1, Ankrd23, Myf6, LOC100910104, Ttn, Homer1, Asb2, Ppp2r3a, Akap6, Tnni1, Tnnc1, Vgll2, Itga7, Trim72, Mypn, Chrnd, Tmod4, Rbm24, Lmod3, Cryab, S100b, Stac3, Arntl, Rxrg, Smtnl1, Rbp4, Tbx1, Ankrd2, Capn3, Cav3, Myh7, Ankrd1, Bves, Mylk2, Popdc2, Ldb3, Ky*
GO:0003012	Muscle system process	2.53E-29	*Ank2, Atp1b1, Myl1, Myl2, Myod1, Myoc, Col14a1, Myl4, Map2k6, Actc1, Actn3, Tead1, Slc25a4, Homer1, Chrnb1, Ttn, Akap6, Cacna1s, Mlip, Tnni1, Mybpc1, Tnnc1, Scn1b, Myh8, Myl3, Tnnc2, Hspb6, Trim72, Jsrp1, Scn4b, Acta2, Ryr1, Tnni2, Chrnd, Tmod4, Lmod1, Lmod3, Myom2, Myh14, Myh11, Myh3, Lmcd1, Stac3, Hrc, Myh2, Actn2, Pgam2, Fxyd1, Mylpf, Tnnt1, Sgca, Myh7, Cav3, Myog, Mb, Trim63, Mybpc2, Mylk2, Myom1, Casq2, Cmya5, Atp1a2*
GO:0006936	Muscle contraction	2.26E-24	*Ank2, Atp1b1, Myl1, Myl2, Myl4, Map2k6, Actc1, Actn3, Homer1, Chrnb1, Ttn, Akap6, Cacna1s, Tnni1, Mybpc1, Tnnc1, Scn1b, Myh8, Myl3, Hspb6, Tnnc2, Jsrp1, Scn4b, Acta2, Ryr1, Tnni2, Chrnd, Tmod4, Lmod1, Lmod3, Myom2, Myh14, Myh11, Myh3, Stac3, Myh2, Actn2, Pgam2, Fxyd1, Mylpf, Tnnt1, Sgca, Cav3, Myh7, Mb, Trim63, Mybpc2, Mylk2, Casq2, Myom1, Atp1a2*
GO:0014706	Striated muscle tissue development	3.89E-22	*Jph2, Myl2, Myod1, Col14a1, Sema3c, Actc1, Sgcg, Slc25a4, Myf6, Smyd1, LOC100910104, Homer1, Ttn, Asb2, Akap6, Cacna1s, Acadm, Neurl1, Tnni1, Vgll2, Tnnc1, Myl3, Itga7, Six1, Klhl41, Wnt5a, Sgcb, Ryr1, Chrnd, Nr4a1, Myh14, Meox2, Myh11, S100b, Eya1, P2rx5, Ndufv2, Stac3, Arntl, Srpk3, Rxrg, Klhl40, Rbp4, Tbx1, Mylpf, Ankrd2, Xirp2, Cav3, Myh7, Eln, Ankrd1, RGD1309821, Myog, Nexn, Bves, Mylk2, Popdc2*
GO:0007517	Muscle organ development	5.09E-22	*Jph2, Myl2, Myod1, Col14a1, Actc1, Fzd1, Sgcg, Slc25a4, Myf6, Smyd1, Homer1, Ttn, Asb2, Akap6, Cacna1s, Neurl1, Tnni1, Vgll2, Tnnc1, Myl3, Itga7, Trim72, Six1, Klhl41, Wnt5a, Sgcb, Ryr1, Chrnd, Cryab, Nr4a1, Myh14, Meox2, S100b, P2rx5, Stac3, Arntl, Srpk3, Rxrg, Klhl40, Smtnl1, Jph1, Rbp4, Tbx1, Mylpf, Ankrd2, Sgca, Xirp2, Cav3, Myh7, Eln, Ankrd1, RGD1309821, Myog, Mylk2, Ky*
GO:0060537	Muscle tissue development	8.94E-22	*Jph2, Myl2, Myod1, Col14a1, Sema3c, Actc1, Sgcg, Slc25a4, Myf6, Smyd1, LOC100910104, Homer1, Ttn, Asb2, Akap6, Cacna1s, Acadm, Neurl1, Tnni1, Vgll2, Tnnc1, Myl3, Itga7, Six1, Klhl41, Wnt5a, Sgcb, Ryr1, Chrnd, Nr4a1, Myh14, Meox2, Myh11, S100b, Eya1, P2rx5, Ndufv2, Stac3, Arntl, Srpk3, Rxrg, Klhl40, Rbp4, Tbx1, Mylpf, Ankrd2, Xirp2, Cav3, Myh7, Eln, Ankrd1, RGD1309821, Myog, Itga8, Nexn, Bves, Mylk2, Popdc2*
GO:0055001	Muscle cell development	1.00E-18	*Ank2, Myl2, Myod1, Col14a1, Actc1, Sgcg, Slc25a4, Myf6, Ankrd23, Flnc, LOC100910104, Homer1, Chrnb1, Ttn, Akap6, Cacna1s, Klhl41, Mypn, Sgcb, Ryr1, Tmod4, Lmod3, Lmod1, Myh11, Stac3, Klhl40, Capn3, Cav3, Myog, RGD1309821, Cfl2, Nexn, Bves, Casq2, Popdc2, Ldb3*
GO:0042692	Muscle cell differentiation	2.25E-18	*Ank2, Myl2, Myod1, Col14a1, Actc1, Mamstr, Sgcg, Csrp2, Slc25a4, Myf6, Ankrd23, Smyd1, Flnc, LOC100910104, Homer1, Chrnb1, Ttn, Asb2, Akap6, Cacna1s, Acadm, Bin1, Trim72, Six1, Klhl41, Mypn, Sgcb, Ryr1, Rbm24, Tmod4, Lmod3, Lmod1, Myh11, Lgals1, Stac3, Trdn, Klhl40, Tbx1, Ppapdc3, Ankrd2, Capn3, Cav3, Nmrk2, Ankrd1, RGD1309821, Myog, Cfl2, Itga8, Nexn, Bves, Casq2, Popdc2, Ldb3*
GO:0006091	Generation of precursor metabolites and energy	3.04E-18	*Sdhd, Grb10, Pgm1, LOC685596, Sucla2, Pkm, Ndufc2, Gpd1, Agl, Pdhb, Acadm, Cat, Prkag3, Ppp1cb, Pfkm, Chchd10, Fh, Sdhb, Ndufa5, Cox8b, Mlxipl, Ndufs4, Pgk1, Gys1, Sdhc, Ndufb8, Idh2, Phka1, Etfdh, Phkg1, Mdh1, Gyg1, RGD2320734, Ndufv2, Phkb, Dlat, Atp5c1, Cisd1, Ppp1r1a, Pgam2, Idh3a, Coq9, Myog, Ndufs1, Gnas, Eno3, Gbas, Sorbs1, Mdh2*
GO:0055002	Striated muscle cell development	5.10E-18	*Myl2, Myod1, Col14a1, Actc1, Slc25a4, Myf6, Ankrd23, Flnc, LOC100910104, Homer1, Chrnb1, Ttn, Akap6, Cacna1s, Klhl41, Mypn, Sgcb, Ryr1, Tmod4, Lmod3, Lmod1, Myh11, Stac3, Klhl40, Capn3, Cav3, Myog, RGD1309821, Cfl2, Nexn, Bves, Casq2, Popdc2, Ldb3*
(B)			
GO:0002376	Immune system process	5.27E-23	*Hck, Oas1b, Coro1a, Tbc1d10c, Siglec10, Il12rb1, Itgam, Dhx58, Olfm4, Prex1, Cd79b, Oasl, Hp, Blk, Unc13d, Csf3r, Syk, Bcl6, Rassf2, Irf8, Ptk2b, Pou2f2, Mx1, Sit1, Nckap1 l, Aim2, Sash3, Plcg2, Mcpt8 l2, Chst3, Tgfbr2, LOC100913063, Isg15, Mx2, Selplg, Np4, Hsh2d, Spi1, RT1-Da, Dock2, C3, Cd74, Nbeal2, Junb, Cxcl13, Ptpn6, Irf7, Ctsg, Ceacam1, Pik3cd, Defa5, Spib, Jak3, Tf, Klhl6, Gfi1, Spn, Inpp5d, Mcpt10, Myo1 g, Siglec15, Milr1, Vpreb1, Cd19, Irf4*
GO:0006955	Immune response	9.26E-18	*Plcg2, Sash3, Hck, Mcpt8l2, Oas1b, LOC100913063, Mx2, Isg15, Np4, Il12rb1, C3, RT1-Da, Dock2, Dhx58, Olfm4, Cd74, Cd79b, Cxcl13, Irf7, Ptpn6, Ctsg, Oasl, Blk, Defa5, Jak3, Unc13d, Klhl6, Syk, Gfi1, Spn, Bcl6, Irf8, Ptk2b, Inpp5d, Mcpt10, Pou2f2, Mx1, Nckap1 l, Vpreb1, Milr1, Myo1g, Aim2, Cd19, Irf4*
GO:0002682	Regulation of immune system process	2.51E-14	*Plcg2, Sash3, Coro1a, Tgfbr2, LOC100913063, Tbc1d10c, Isg15, Il12rb1, Spi1, C3, Dhx58, Olfm4, Cd74, Cd79b, Cxcl13, Irf7, Ptpn6, Ceacam1, Ctsg, Blk, Jak3, Unc13d, Csf3r, Klhl6, Syk, Gfi1, Spn, Bcl6, Rassf2, Ptk2b, Inpp5d, Sit1, Nckap1l, Milr1, Myo1g, Siglec15, Aim2, Cd19, Irf4*
GO:0002520	Immune system development	6.56E-13	*Plcg2, Sash3, Coro1a, Tgfbr2, LOC100913063, Isg15, Siglec10, Spi1, Itgam, C3, Dock2, Prex1, Cd74, Nbeal2, Cxcl13, Junb, Ptpn6, Ceacam1, Blk, Jak3, Spib, Tf, Csf3r, Syk, Spn, Bcl6, Rassf2, Irf8, Ptk2b, Inpp5d, Pou2f2, Nckap1l, Vpreb1, Siglec15, Irf4*
GO:0048534	Hematopoietic or lymphoid organ development	8.85E-13	*Plcg2, Sash3, Coro1a, Tgfbr2, LOC100913063, Isg15, Siglec10, Spi1, Itgam, Dock2, Prex1, Cd74, Nbeal2, Cxcl13, Junb, Ptpn6, Ceacam1, Blk, Jak3, Spib, Tf, Csf3r, Syk, Spn, Bcl6, Rassf2, Irf8, Ptk2b, Inpp5d, Pou2f2, Nckap1l, Vpreb1, Siglec15, Irf4*
GO:0046649	Lymphocyte activation	1.21E-12	*Pik3cd, Plcg2, Sash3, Blk, Jak3, Coro1a, Unc13d, Tgfbr2, LOC100913063, Tbc1d10c, Syk, Hsh2d, Spn, Il12rb1, Spi1, Bcl6, Itgam, Dock2, Ptk2b, Inpp5d, Prex1, Cd74, Pou2f2, Sit1, Nckap1l, Vpreb1, Ptpn6, Irf4, Ceacam1*
GO:0045321	Leukocyte activation	2.23E-12	*Pik3cd, Plcg2, Sash3, Blk, Jak3, Coro1a, Unc13d, Tgfbr2, LOC100913063, Tbc1d10c, Syk, Selplg, Hsh2d, Spn, Il12rb1, Spi1, Bcl6, Itgam, Dock2, Ptk2b, Inpp5d, Prex1, Cd74, Pou2f2, Sit1, Nckap1l, Vpreb1, Milr1, Ptpn6, Irf4, Ceacam1*
GO:0002252	Immune effector process	3.54E-12	*Plcg2, Sash3, Oasl, Oas1b, Jak3, Unc13d, Mx2, Isg15, Syk, Spn, Il12rb1, Bcl6, Dock2, C3, Ptk2b, Dhx58, Inpp5d, Cd74, Pou2f2, Mx1, Irf7, Milr1, Ptpn6, Myo1g, Aim2, Irf4, Ctsg, Ceacam1*
GO:0002521	Leukocyte differentiation	4.09E-12	*Plcg2, Sash3, Blk, Spib, Jak3, Tf, Coro1a, Tgfbr2, LOC100913063, Syk, Spn, Spi1, Rassf2, Bcl6, Itgam, Dock2, Ptk2b, Inpp5d, Prex1, Cd74, Junb, Pou2f2, Nckap1l, Siglec15, Ptpn6, Irf4*
GO:0002684	Positive regulation of immune system process	5.20E-12	*Plcg2, Sash3, Blk, Jak3, Coro1a, Unc13d, Tgfbr2, LOC100913063, Klhl6, Isg15, Syk, Gfi1, Spn, Il12rb1, Bcl6, C3, Ptk2b, Dhx58, Inpp5d, Cd74, Cd79b, Cxcl13, Nckap1l, Irf7, Myo1g, Ptpn6, Aim2, Irf4, Cd19, Ceacam1, Ctsg*

**Figure 1:**
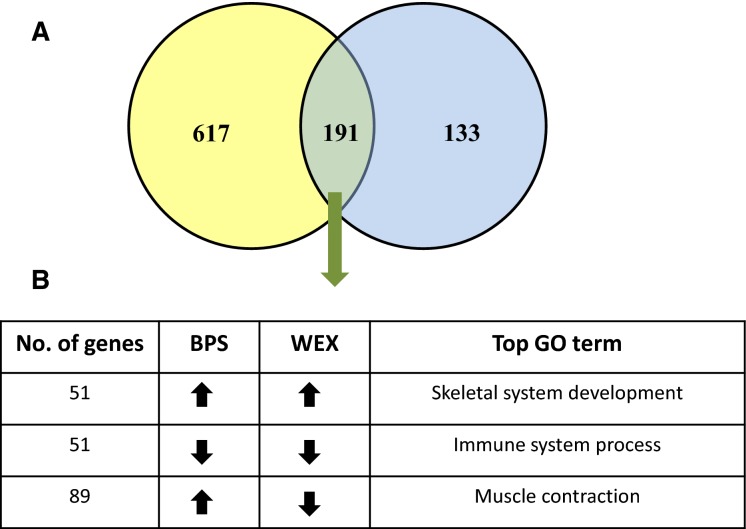
Venn diagram showing the distribution of genes that responded to BPS and WEX exercises. (A) 617 genes responded solely to BPS exercise while 133 genes were differentially expressed only in WEX. 191 genes were differentially expressed both in BPS and WEX compared to the CON group. (B) Among the 191 genes that were differentially expressed both in BPS and WEX, 51 were up-regulated and 51 were down-regulated in both exercise groups compared to the control. The behavior of the remaining 89 genes depended on the exercise imposed. While they were up-regulated in the BPS group, the same set of genes was down-regulated in the WEX group. The up-regulated gene list was enriched for terms associated with skeletal system development. The down-regulated gene list was enriched for immune system processes and the remaining 89 genes were involved in muscle contraction. CON, Control; BPS, Bipedal Stance; WEX, Wheel Exercise.

There were 191 gene transcripts that were differentially regulated in both forms of exercise. Within this common set of gene transcripts, 51 were up-regulated in both conditions and were enriched for GO terms associated with skeletal system development. Fifty-one transcripts were down-regulated in both forms of exercise and were enriched for immune system processes. The remaining 89 gene transcripts were enriched for GO terms related to muscle contraction. The expression changes of these 89 gene transcripts were directionally dependent upon the exercise modality: specifically, these 89 transcripts were up-regulated in the BPS and down-regulated in the WEX group (Fig.1).

In order to determine if any of the common 191 differentially regulated transcripts were influenced by the magnitude of the exercise performed, the WEX animals were divided into two subgroups based on their individual total running distance within a monitored 36 hour period (D_33_). The mean (±SEM) distances run by the low (2130 ± 575.55 m) and high (3888.04 ± 825.9 m) running subgroups were significantly different (*P*-value: 0.04). Differential gene expression analysis between the high and low running subgroups identified six transcripts (*LOC100360843, Ankrd2, Myh7, Myl3, Myoz2, and Tnnt1*) that were significantly changed in the high, and two transcripts (*Fkbp5* and *Ccl21*) that were significantly changed in the low running subgroups.

### Pathway analysis

Ingenuity pathway analysis revealed that the WEX-affected transcripts were significantly enriched in pathways for epithelial adherens junction signaling, calcium signaling, integrin linked kinase (ILK) signaling, cellular effects of sildenafil, and remodeling of epithelial adherens junction signaling (Table4). By contrast, BPS-affected transcripts were significantly enriched in calcium signaling, ILK signaling, cellular effects of sildenafil, oxidative phosphorylation, and mitochondrial dysfunction pathways (Table4).

**Table 4:** Canonical pathways enriched for differentially expressed transcripts in BPS and WEX rats. Enrichment was determined using IPA. The P-value was calculated using the Fisher’s exact test. The ratio gives an indication of the number of differentially expressed transcripts compared to the total number of genes in the dataset that correspond to a canonical pathway

	BPS				WEX		
No	Canonical pathway	*P*-value	Ratio	No	Canonical pathway	*P*-value	Ratio
1	Oxidative phosphorylation	1.66 ^*^ 10^−31^	40/86 (0.465)	1	Epithelial adherens junction signaling	1.07 ^*^ 10^−04^	10/136 (0.074)
2	Mitochondrial dysfunction	2.21 ^*^ 10^−25^	44/144 (0.306)	2	Calcium signaling	1.24 ^*^ 10^−04^	11/166 (0.066)
3	Calcium signaling	8.83 ^*^ 10^−10^	28/166 (0.169)	3	ILK signaling	1.62 ^*^ 10^−04^	11/171 (0.064)
4	Cellular effects of sildenafil	1.87 ^*^ 10^−08^	22/123 (0.179)	4	Cellular effects of sildenafil	2.45 ^*^ 10^−04^	9/123 (0.073)
5	ILK Signaling	3.27 ^*^ 10^−08^	26/171 (0.152)	5	Remodeling of epithelial adherens junctions	5.67 ^*^ 10^−04^	6/61 (0.098)

**Figure 2:**
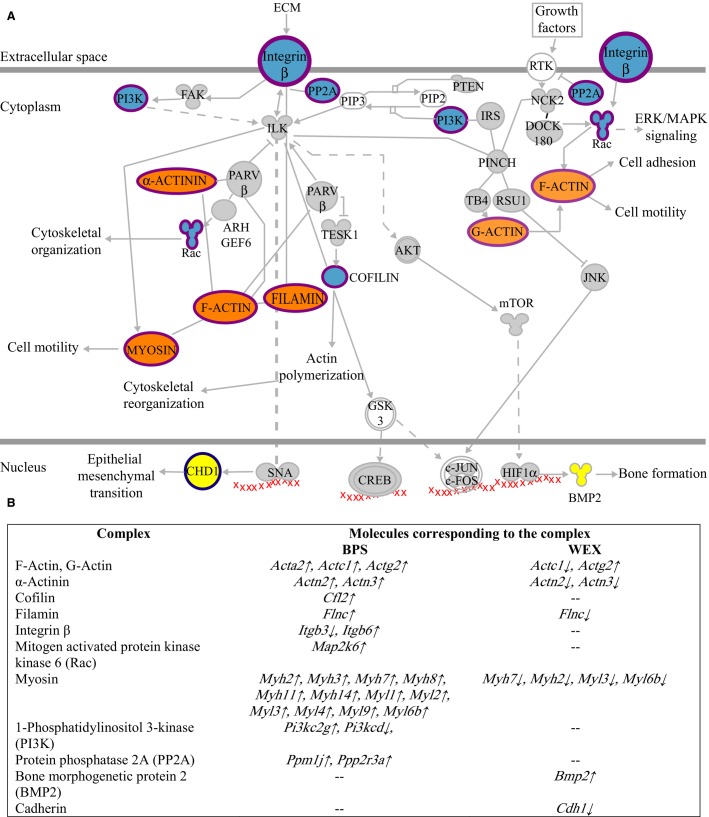
Molecular complexes involved in the integrin-linked kinase signaling pathway responded to BPS and WEX treatments. (A) Schematic of the integrin-linked kinase signaling pathway. Complexes highlighted in blue responded to BPS exclusively. Complexes highlighted in yellow responded to WEX exclusively. Complexes highlighted in orange responded to both treatments. (B) Individual members of the complexes that responded to the different exercises are listed in the table. The direction of the arrows next to the genes indicates up-regulation (↑) or down-regulation (↓) of the corresponding transcript.

Ca^2+^, ILK and sildenafil pathways responded to both BPS and WEX. However, the genes that responded to BPS and WEX within these pathways exhibited exercise-dependent changes in transcript levels. For example, the transcript levels of nine genes in the ILK signaling pathway responded to both treatments (Fig.2). Of these, eight transcripts were up-regulated in BPS and down-regulated in WEX. Only one gene transcript (*Actg2*) was up-regulated in both treatments (Fig.2).

**Figure 3:**
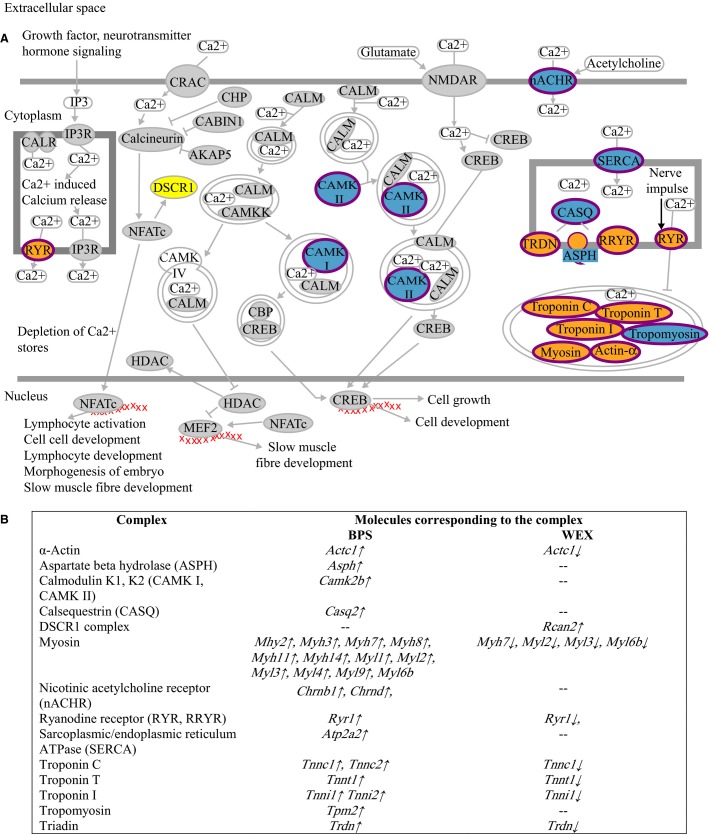
Molecular complexes involved in the calcium signaling pathways responded to BPS and WEX treatments. (A) Schematic of the calcium signaling pathway. Complexes highlighted in blue responded to BPS exclusively. Complexes highlighted in yellow responded to WEX exclusively. Complexes highlighted in orange responded to both treatments. (B) Individual members of the complexes that responded to the different exercise treatments are listed in the table. The direction of the arrows next to the genes indicates up-regulation (↑) or down-regulation (↓) of the corresponding transcript.

Transcripts associated with Ca^2+^ signaling pathways also exhibited exercise-dependent changes (Fig.3) for 10 genes. Again, these genes showed diametrically opposite responses to the exercise modalities being up-regulated in BPS, and down-regulated in WEX (Fig.3).

## Discussion

We determined the in vivo effects of two simple exercises on gene transcript levels within the femoral mid-diaphysis of prepubertal male rats on D36 following 15 days of voluntary exercise. We used a 15 day regimen of voluntary mild exercise in pre-pubertal male rats because: (1) almost all literature concerning exercise in the young and its effect on adult musculo-skeletal system phenotype deals with high exercise loads associated with competitive sport; (2) the regime we implemented did not cause a stress response (Sontam et al. 2015); (3). the 15 day period of exercise avoided the highly variable time of onset, effects, and endocrine status of animals becoming pubertal during the experiment; and (4) the use of male rats avoided the highly variable physical activity that is typical of female rats. The main limitation of our experiment was that it could not be established that such mild exercise would have phenotypic effects on bone and other tissues later in life. That gene transcript levels were so different between exercise groups was very notable.

Exercise induces a number of physiological responses within the body such as changes in blood flow and oxygenation (Albouaini et al. 2007), an endocrine response (Price et al. 2011), and increased muscle and tendon forces (Burr 1997) all of which, in concert, influence the overall response of bone to exercise (Meakin et al. 2014). This is particularly relevant to our findings as the cortical bone of the mid-diaphysis is a complex heterogeneous tissue consisting of marrow, vasculature, epithelium, and nerves within an extracellular matrix that is mineralized to a variable degree. The changes we observed in transcript levels cannot be attributed to specific cell types within the bone. However, the data in the current study demonstrate some of the complex morphological, architectural, and gene expression changes that occur in bone in response to the mechanical loading associated with growth and physical activity.

Both BPS and WEX have previously been shown to elicit an osteogenic response in the rat (Newhall et al. 1991; Rosa et al. 2010). However, our results show significant modulation of transcript levels according to exercise modality. The two exercise modalities differed in their gene expression signatures with respect to: 1) the number of genes that responded; (2) the directionality of response (up- or down-regulated); and (3) the magnitude of response. At the same time, there were changes to common genes and signaling pathways. Crucially, the different exercise modalities had opposite effects on key components within the ILK and Ca^2+^ signaling pathways that are two prominent mechanotransduction pathways involved in bone’s adaptation to exercise (Papachristou et al. 2009; Marie et al. 2014). Therefore, while it remains possible that these signals are hormonal, the differences in transcript levels for mechano-responsive molecules (*e.g*. integrin *β*) indicate that the strain magnitude, strain frequency, and fluid shear forces may signal the exercise modality (Milgrom et al. 2000).

Both ILK and Ca^2+^ signaling pathways are known for their role in mechanotransduction (Papachristou et al. 2009; Marie et al. 2014). Integrins are heterodimeric transmembrane adhesion proteins that have the ability to relay signals between the extracellular matrix and the cytosol. Activation of the heterodimeric complex leads to its association with and reorganization of the actin cytoskeleton. This reorganization may influence osteoblastic gene expression through cytoskeletal-nucleoskeletal linkages (Pavalko et al. 1998). The actin cytoskeleton may also function as a scaffold for the translocation of signaling molecules from the membrane to the nucleus (Pavalko et al. 1998). Additionally, integrin activation leads to the modulation of activity of focal adhesion kinase (FAK) and ILK, which are involved in several signal transduction pathways in bone cells (Novak et al. 1998; Marie et al. 2014).

Ca^2+^ is a well-known second messenger in many cell types and plays a signaling role in bone metabolism. For example, in osteoclasts RANKL activation of RANK increases intracellular Ca^2+^ which initiates downstream signaling processes that result in osteoclast differentiation (Seales et al. 2006). Increases in intracellular Ca^2+^ in response to fluid shear stress are vital for actin stress fiber formation and subsequent gene expression changes in osteoblasts (Chen et al. 2000). In osteocytes, increased intracellular Ca^2+^ is necessary for pulsating fluid flow-induced prostaglandin E2 (PGE2) release (Ajubi et al. 1999). Additionally, many of the transcripts that IPA identified to be part of sildenafil pathway play an integral role in calcium signaling. For example, the L-type and voltage-gated calcium channels, GNAS locus, and phospholipase C (PLC) are all necessary for calcium-mediated signaling in bone (Zayzafoon 2006) and must, in the present context, be considered to be a part of the Ca^2+^ signaling pathways.

Genes traditionally associated with muscle development and function responded to one or both exercises. This is consistent with earlier studies that have shown down-regulation of muscle genes within rat ulna in response to axial loading (Mantila Roosa et al. 2011). Moreover, Paic et al. identified elevated expression of muscle-related transcripts in osteocytes compared to osteoblasts (Paic et al. 2009). Therefore, it is possible that our observations reflect a change in the ratio of osteocytes relative to osteoblasts in response to BPS and WEX.

We hypothesized that mild to moderate exercise will elicit measurable changes in the gene expression profiles in the mid-diaphysis of prepubertal male rats whose bone micro-architecture and morphology showed minor changes in response to exercise. We identified and characterized several transcripts whose expression levels were affected by the exercise undertaken. The observation that the two exercise modalities elicited a diametrically opposite response for a subset of transcripts is particularly relevant given the perception that physical exercise promotes long and short-term bone health. Indeed, our results are consistent with earlier observations that the form and intensity of the exercise is critical for the bone-specific response that is elicited (e.g. [Milgrom et al. 2000]). Direct comparisons regarding the activity type and activity cycles and therefore the strain magnitude and frequency required to elicit these changes are not currently possible, as there are no biomechanical models for these exercises in the rat. Moreover, determination of the actual exercise-dependent changes in the strain environment of bone cannot currently be empirically determined (Meakin et al. 2014). We contend that future studies should characterize the exercise-dependent changes that occur at specific regions and to specific cells within bones, and at different stages of growth and development, in order that the full benefits of exercise can be harnessed to improve or maintain bone health.

## Conflict of Interest

The authors declare no conflicts of interest, financial or otherwise, regarding the publication of this article.
